# Shaping public opinion on the issue of childbirth; a critical analysis of articles published in an Australian newspaper

**DOI:** 10.1186/1471-2393-11-47

**Published:** 2011-06-28

**Authors:** Meredith J McIntyre, Karen Francis, Ysanne Chapman

**Affiliations:** 1 School of Nursing & Midwifery, Monash University, Peninsula Campus, Frankston, Australia; 2 School of Nursing & Midwifery, Monash University, Gippsland Campus, Churchill, Australia; 3 School of Nursing & Midwifery, Central Queensland University, Mackay Campus, Australia

## Abstract

**Background:**

The Australian government has announced a major program of reform with the move to primary maternity care, a program of change that appears to be at odds with current general public perceptions regarding how maternity care is delivered.

**Methods:**

A critical discourse analysis of articles published in 'The Age', a newspaper with national distribution, subsequent to the release of the discussion paper by the Australian Government in 2008 was undertaken. The purpose was to identify how Australian maternity services are portrayed and what purpose is served by this representation to the general public.

**Results:**

Findings from this critical discourse analysis revealed that Australian maternity services are being portrayed to the general public as an inflexible outdated service struggling to meets the needs of pregnant women and in desperate need of reform. The style of reporting employed in this newspaper involved presenting to the reader the range of expert opinion relevant to each topic, frequently involving polarised positions of the experts on the issue.

**Conclusions:**

The general public are presented with a conflict, caught between the need for changes that come with the primary maternity model of care and fear that these change will undermine safe standards. The discourse; 'Australia is one of the safest countries in which to give birth or be born, what is must be best', represents the situation where despite major deficiencies in the system the general public may be too fearful of the consequences to consider a move away from reliance on traditional medical-led maternity care.

## Background

The Australian government has announced a major program of reform with the move to primary maternity care in line with the recommendations of the national review of maternity services released in 2009. The reform represents a program of change that appears to be at odds with current general public perceptions regarding how maternity care is delivered. Primary maternity services offer women continuity of primary carer with a midwife or general practitioner, reserving obstetric services for only those in clinical need [1,2]. Australia is a society that has embraced the introduction of high technology across all aspects of life including childbirth, a situation reflected in the number of healthy Australian women who elect private obstetric services in the absence of clinical risk [3]. There is no longer need to give birth to a child with abnormalities as we have diagnostic tests to protect us. Reproductive technologies have rescued women from the consequences of infertility. Women do not have to suffer the pain of labour as medical intervention makes this unnecessary, in the same way we no longer expect to experience pain in other areas of our lives [4]. In 2008 the majority of Australian women (96.9%) gave birth in either obstetric-led public or private hospitals [3], reflecting a widespread acceptance that birth needs to be medically managed. A small but vocal group of women chose to give birth in non-obstetric services such as birth centres (2.2%) or home birth (0.9%) in the care of experienced midwives [3]; services based on a belief that giving birth is a seminal life event for women [5] and not a medical event. Core values underpinning these birthing options; a strong belief in women's ability to birth with minimal intervention, continuity of care and women's right to informed choice are shared by the proposed maternity care reforms. Consistent with these values, the agenda for change in maternity service delivery involves a move away from reliance on expensive specialist medical services for all pregnant women in order to reserve these valuable services for those women who need them [2].

Although many women expect childbirth to be a potentially non-affirming event steeped in pain and fear [6], giving birth safely is considered to be the most important thing a mother does for her child and safeguarding that child is implicit in this responsibility. A reflection of our increasingly risk-averse society pregnant women are reporting increasing levels of fear [7-9] related to the possibility of something going wrong and concern for the wellbeing of their baby. Women, it is argued, want to be able to avoid future regret [4] in the belief that a perfect child is more important than the perfect birth [10]. They place trust in their care providers to keep them safe, willingly doing whatever they have been led to believe might help secure a perfect healthy baby [10].

Many women know little about maternity care prior to becoming pregnant for the first time and are guided by the advice of health professionals, family and friends or the media when choosing the maternity care that best suits their needs. Information presented in the media can be highly persuasive as illustrated by two recent high profile exposès on maternity care including the US based DVD 'the business of being born' [11] and the Australian based book 'the birth wars' [12]. 'The business of being born' promotes the benefits of midwife -led home birth arguing that obstetrics is a money making business engaging in practices that are business enhancing rather than being in the best interests of the health and wellbeing of women [11]. "The birth wars' explores the sometimes acrimonious contest between the medical and midwifery professions for the control of uncomplicated birth [13] and the negative consequences to pregnant women and their babies caught in the middle [12]. Both of these exposès cast doubt on the motivation behind modern maternity care and when combined are capable of undermining the general public's confidence in the service.

Implementation of the primary maternity care reforms represents a major change to the way maternity care has been traditionally delivered in Australia. Successful implementation requires the support of the general public for whom the service is intended. One source of influence on general public opinion is the newspaper media and the news feeds that spin off to television and radio from feature articles of interest. The powerful use of language in a headline is used to attract general public attention to reports on controversial topics affecting Australian maternity care. This study examines the newspaper articles published in 'The Age' newspaper subsequent to the release of the Government discussion paper 'improving maternity services in Australia' in 2008 [1] that heralded the national review of maternity services consultation process.

## Methods

### Research design

A critical discourse analysis of articles published in the 'The Age' subsequent to the release of the discussion paper by the Australian Government in 2008 [1] was undertaken commencing November 2008 through to January 2011. The purpose was to identify how Australian maternity services are portrayed and what purpose is served by this representation to the general public. 'The Age' newspaper was chosen as the data source for this study as it has national distribution and regularly features news articles that report controversial issues particularly in the area of maternity care and childbirth. The newspaper also sources news articles of interest from syndicated sources both nationally and internationally. Discourse analysis enables the examination of text in the form of newspaper articles [14]. Textual context of newspaper articles do not represent reality, rather they create a version of it. Each article represents a version of reality which is designed to produce effects, these versions can vary depending on the situation being portrayed in each article. Discourse according to Foucault (1972) actively constitutes or constructs society using knowledge and power [15]. A discourse can be viewed as a total system of knowledge that makes a multitude of true statements possible whilst articulating a particular truth and then maintaining the effects of that truth [16]. Pertinent to this study are the questions; what can be known and what can be said on the topic of maternity services in Australia and the spokespersons created within the discourse.

Critical analysis of discourse as a methodology was chosen for this study because language takes on greater significance in the arena of providing and consuming services [17]. Obstetrics and midwifery have a long history of professional power struggles whilst the consumer groups also contain polarised elements making an analysis of language used to influence public attitudes an appropriate choice. Critical analysis of discourse provides insight into how bodies of knowledge are used to influence public perception [18]. The ability to generate interpretive claims with regard to the desired consequences of controlling knowledge [19], combined with the ability to critically examine power relationships constituted by discourse [20] are relevant to this study.

### Theoretical Framework

Critical social theory has greatly influenced the development of CDA bringing together a wide variety of critical social theories including the way power is conceptualized [21]. Through his work Foucault has influenced the understanding that discourse is 'an instrument in the social construction of reality'[17]. CDA views discourse as central to the functioning of power in social processes and the reproduction of power in a given situation, and to understand the processes of power and how these processes use discourses to achieve power. It is crucial that language is not analyzed out of context, but is situated within the specific context of social practices of which it is a part [22]. This necessitates an approach that takes in the broad context of the text in addition to the specific words used.

### Ethical Considerations

All research data consisted of articles featuring the topics of maternity services or childbirth published in 'The Age' newspaper. Articles were obtained from the public domain, accessed through an online search of 'The Age' newspaper website. Personal information was not retrieved from any source and there was no participant involvement.

### Data Collection

A search was conducted on 'The Age' newspaper website for articles including the search terms; maternity services, birth and childbirth. A total of thirty-eight articles were indentified and included in the study. The title of each article including date and section of publication is supplied in Table 1.

**Table 1:** Articles published in the 'Age" newspaper (URL: http://www.theage.com.au/)

Date	Title	Section
January 2, 2011	*There's no shame in being too posh to push*	National News (1)
December 6, 2010	*Babies by the dozen but medic says home birth too risky*	Victoria(2)
September 22, 2010	*Too posh to push? Ask my doctor*	Lifestyle (3)
August 25, 2010	*Midwife-led team to replace doctors*	Breaking News (4)
August 11, 2010	*Pregnancies swell public system as costs rise*	National News (5)
July 1, 2010	*Home births triples neonatal death risk: study*	Lifestyle (6)
June 20, 2010	*Hard Labour*	Victoria (7)
May 30, 2010	*Caesareans take toll on babies*	National News (8)
May 9, 2010	*Public hospitals neglecting postnatal care*	Victoria (9)
April 7, 2010	*Easing labour pain naturally*	Lifestyle (10)
March 5, 2010	*Concerns for bush maternity services*	National News (11)
January 21, 2010	*One born every minute*	Opinion (12)
January 18, 2010	*Obstetricians urge caution on homebirths*	Breaking News (13)
January 9, 2010	*Never again in a public hospital*	National News (14)
January 9, 2010	*No perfect system*	National News (14.1)
January 9, 2010	*It's not all gloom*	National News (15)
January 9, 2010	*Motherhood's trauma*	National News (16)
January 9, 2010	*A cry for help*	National News (17)
January 2, 2010	*Mother care; it's like herding yards*	National News (18)
January 2, 2010	*A child is born*	National News (19)
January 2, 2010	*Mothers' views of maternity: public v private*	National News (20)
January 2 2010,	*Mothers deliver verdict on maternity hospitals*	Opinion (21)
December 23, 2009	*Home-birth boost for expectant mothers*	National News (22)
September 5, 2009	*Special delivery*	Good weekend (23)
May 13, 2009	*Rural doctors welcome new midwife powers*	Breaking News (23.1)
April 16, 2009	*Hospital, home births 'no difference'*	Breaking News (24)
April 9, 2009	*A home birth is not a safe birth*	Lifestyle (25)
April 7, 2009	*Rural maternity services 'face crisis'*	National News (26)
April 6, 2009	*Overhaul maternity system, doctors say*	Breaking News (27)
March 22, 2009	*Home deliveries*	National News (28)
February 26, 2009	*Jump in birth problems call for better care*	National News (29)
February 20, 2009	*Maternity units 'herding yards'*	National News (30)
January 7, 2009	*Anger over 'third world' pregnancy death*	National News (31)
January 6, 2009	*Fear drives growth in caesareans: expert*	National News (32)
December 13, 2008	*When it comes to childbirth, it's not always hail caesar*	National News (33)
December 10, 2008	*Maternity care hit by shortages*	National News (34)
December 9, 2008	*Respect choices, maternity review told*	National News (35)
November 30, 2008	*In safe hands*	National News (36)

### Data Analysis

Fairclough's approach to CDA links textual and sociological analysis in a way that foregrounds issues of power, resistance and identity [14]. In this regard the language and practices of key stakeholders presented in the newspaper media can be examined for the purpose of understanding how these shape and limit the general public perception of the issue in question. This process has the ability to reveal the operation of power within a text. The analysis of relations of power and constructions of 'truths' in this study allowed the researcher to identify stimulus that explain changed roles, relationships and institutional practices associated with the primary maternity care reform agenda. Fairclough (1992) describes three aspects that need to be considered when looking at a text in the process of CDA. In order these are: the context in which the text is produced; the way it is received and the details of the text itself [23]. In this study these aspects of the discourse were examined using a three step approach; the socio-cultural level; the discourse practice level and the textual level [14].

#### Socio-cultural level

Analysis undertaken at the socio-cultural level refers to an analysis of the social context in which the text under investigation is produced. Texts were analysed for each key stakeholder group presented in the news article to uncover the unspoken and unstated assumptions implicit within them which have shaped the very form of the text in the first place [24]. This level of analysis in our study allowed the text to be assessed within the environment in which it was created [23]. Macro level discourses were identified and manually coded during this descriptive analysis of text [23].

#### Discourse practice level

Analysis undertaken at the level of discourse practice is concerned with the production, distribution and consumption of the text [23]. In this study the nature of the discourse practice relied on the interaction between the various socio-cultural practices present and in which the discourse was situated. Analysis includes the history and practices surrounding the textual medium through which the text under analysis is presented [23]. Micro level discourses were identified, including the position promoted by the discourse and by whom. A continuation of the manual coding process undertaken in Step 1 of the analysis was employed for each key stakeholder group [23].

#### Textual level

Analysis at the textual level is concerned with the minutiae of the text such as how it is formed and what particular vocabulary and style is used in order to produce meaning [23]. This study employed a deductive approach using the codes identified through content analysis in the first two steps. The codes were searched for instances where power and knowledge were present in the discourse, by whom and how this power was being used [14].

#### Methodological rigour

Critical discourse analysis is an interpretive process and acknowledges the multiple interpretations that can emerge from the text. The source of text included in the study has been explained and matched to the aims of the research. Sufficient quantities of text have been included in the analysis to capture all that can be known or said on the topic. Rigour in this study was enhanced through careful selection of text ensuring the discourses of all key stakeholders were represented in the analysis, as illustrated in Table 1. To strengthen interpretive claims a number of verbatim text have been provided to support each finding.

## Results

Critical analysis of articles published in 'The Age' generated a number of discourses exerting influence on general public opinion. By far the majority of articles were concerned with sensationalising the deficiencies of maternity services. The articles featured attention grabbing headings, highlighting issues pertaining to the state of maternity services, supplemented by a range of expert opinions including the relevant government authority, medical or obstetric experts, Australian College of Midwives (ACM) and consumer groups.

Expert consumer opinion was sought by 'The Age' from organisations such as maternity coalition or future families, advocates for primary maternity care reform supporting natural birth options. These organisations are well organised publicity machines promoting their position on natural childbirth [25]. What was found to be missing from the perspective of the newspaper was a similar organisation representing the position of women satisfied with obstetric care. To address this deficit 'The Age' newspaper undertook a survey of women who had given birth in the past five years through the Fairfax essential baby website, attracting 2,792 participants who were asked specific questions regarding their experience of maternity care.

### The state of the maternity services

In the process of sensationalising the deficiencies of maternity services, a number of articles extensively reported on the findings of a survey of 2792 mothers highlighting the traumatic or unsatisfactory experiences of women giving birth. Discourses generated from a critical analysis of that study included; 'the interests of women and their babies must be the focus of maternity care, not the system and those who work in it', 'next please, feeling depersonalised in the queue'. These discourses imply that current maternity services operate in the interests of the institution and maternity care professions and not in the interests of the women receiving care. The woman's personal experience and special needs are dismissed in an overstretched system providing a 'one size fits all service'. Selected statements illustrating the meanings of these discourses included:

*'Women feeling like cattle being pushed through herding yards that put both their own and their babies' lives at risk. Hundreds of women felt that both public and private maternity wards are overcrowded, needed more midwives, performed too many unnecessary medical interventions and did not provide enough non-drug options for pain relief (quote article 18)*.

*'Nothing could have prepared me for my horrible birth experience - "herding yards does not go nearly far enough in describing the way the hospital treats new mothers and babies (quote article 14)*.

The safety of overcrowded and understaffed metropolitan maternity services is called into question in the majority of these news articles. It has been argued that maternity care in Australia is delivered in an outmoded 'hospital nursing' model [26] that has been proven over the years to be inflexible in meeting the complex psychosocial and emotional needs of women having babies [27,28]. Constraints associated with running a hospital contribute to the provision of fragmented maternity care to women who need the support of continuity of care at this important time in their lives [25,29]. Conflicting information and advice is a feature of current maternity services with staffing arrangements that cannot secure a known caregiver. A constant stream of unknown carers undermines trust women have in the care received and decisions being made. Discourses generated from this study include; 'women feeling vulnerable in the care of a parade of strangers', 'women expected to place blind trust in those who know nothing about me and still feel safe'. Selected statements illustrating the meanings of these discourses included:

*'A large public hospital means a huge variation in staff on different shifts, which leads to inconsistent care and the danger of falling through the cracks' (quote article 17)*.

*'The most concerning theme centres on the apparent shortage of resources in public hospitals, leaving many women feeling as if they and their infants are being exposed to unacceptable risk' (quote articles 18 & 21)*.

*'Because of workforce difficulties, care is fragmented. Women might see 12 or 13 diffent people during the pregnancy' (quote article 27)*.

A number of the newspaper articles reported on the findings of the national review of maternity services released in 2009. One of the main messages highlighted in the articles is the need to respect women's right to choose, inferring that women are not being offered choice in the maternity care they receive. Some women in an attempt to investigate childbirth options find themselves labelled by the health professionals, on whom they rely, as being 'difficult' or 'untrusting' [10]. Discourses generated from this study include; 'maternity care options need to be expanded to allow women choice in the care they receive', 'the demonization of women who choose to give birth naturally'. Selected statements illustrating the meanings of these discourses included:

*'Stop treating pregnancy as an illness and respect women's birth choices' (quote article 35)*.

*'Women seduced by the "empowering" idea that only a woman knows how to deliver her child forget that 100 years ago 1 in 10 women died from complications of childbirth and 1 in 10' (quote article 25)*.

*'Women do not have to prove to health professionals that their healthcare choices are good or worthy ones to have the right to decide' (quote article 1)*.

Several newspaper articles question why Australian women are more anxious about their pregnancies despite Australia's safe record in childbirth. The chance of a women dying during childbirth in Australia is remote [3]. Of concern is when high levels of fear are sustained due to women's perception of their own birth risk being out of proportion to actual medical risk [7,30]. Everyone wants a healthy child and women willingly do whatever they have been led to believe might help [10]. Discourses generated from this study include; 'every pregnancy is treated with suspicion until proven otherwise', 'modern medicine knows best and women are best served to listen to the experts'. Selected statements illustrating the meanings of these discourses included:

*'Many women are feeling more, rather than less, anxious about the birth process. Some blame this on our risk-averse society, saying the screens and tests and the increasing level of intervention in birth and pregnancy is geared towards making women fearful. Antenatal care has become antenatal scare' (quote article 7)*.

*'The woman's surgery was scheduled without her consent and she claimed she was bullied by hospital staff, who said her baby could die if she did not follow their advice. This new mother never wanted to birth at home - she wanted the support of the hospital but the system would not provide the care' (quote article 30)*.

*'In one Australian study, 62% of women who had a caesarean section in a private hospital said their health care provider had recommended it' (quote article 1)*.

*'There should be more continuity of care. Knowing your carer and trusting your carer removes the fear from childbirth and fear leads to more interventions' (quote article 19)*.

#### In the rural setting

The plight of rural maternity services was highlighted in a number of articles as a serious deficiency in current maternity service delivery [25], a situation exacerbated by continuing service closures [31]. The inequity experienced by pregnant women living in rural and remote locations forced to travel long distances to access maternity care, is raised as a failure of government to provide essential services to these communities [31-33] placing lives at risk. The deadly consequences associated with insufficient rural maternity services was highlighted by the report of the death of a pregnant Victorian woman. Rural services were compared to third world conditions reinforcing an inequity imbedded in a system that privileges one group over another, usually the more vulnerable group. The discourse identified; 'the lives of mothers and babies in rural communities are placed at risk by widespread closures of maternity services'. Selected statements illustrating the meanings of these discourses included:

*'Since 1996, 40 maternity units had closed across the state and others were near closure. It's reflective of a general shortage of GP's across rural Australia. As a society we have not invested enough in GP proceduralists: doctors who provide obstetric care, anaesthetics or emergency' (quote article 26)*.

*'Urgent action is required to prevent women enduring "roadside births" as they travel hundreds of kilometres to dwindling maternity services in regional and rural Australia' (quote article 34)*.

*'Reopen my local maternity unit. Travelling while in labour is not only uncomfortable, but dangerous. (we only just made it to the hospital before our baby was born' (quote article 19)*.

### Expert opinion on controversial issues

All articles retrieved for this study presented a range of expert opinion on the topic featured, usually including the relevant government authority, medical or obstetric experts, the ACM and consumer groups. Opinions represented a range of views often involving polarised positions on the issue. Consumers and midwives reinforced the position of natural birth and challenged increasing rates of medical intervention. Medicine or obstetrics highlighted the dangers of medically unassisted childbirth while the government defended the proposed maternity service policy direction. The main issue debated in the articles centred on contention surrounding government support for midwifery -led maternity services extending to including controversial home birth trials. As exemplified in the statement:

*'Victorian women will be able to give birth at home - with hospital back up for the first time - under a pilot project starting at three hospitals next year' (quote article 22)*.

The safety record of home birth is reported in a number of newspaper articles as having received a boost with several large studies in recent years reporting no significant difference between planned hospital birth and planned home births, as long as the mothers have access to trained midwives [34,35].

*'A Dutch study of more than 500,000 women reported that planning a home birth does not increase the risks of perinatal mortality and severe perinatal morbidity among low risk women provided they were supported by well trained midwives and a good transportation and referral system' (quote article 24)*.

### Obstetric opinion

Medical or obstetric opinion reported in the newspaper articles reflect the position that they are the guardians of safety in childbirth in the belief that only they have the training and expertise for safe birth outcomes to be achieved [36]. The expert voice in this regard is the president of the Royal Australian and New Zealand College of Obstetrics and Gynaecology (RANZCOG) who reminds readers that current excellent obstetric outcomes are due to high quality medically- led maternity services [37]. A position supported by the secretary general of the Australian Medical Association (AMA) warning that the introduction of non-medically led services will undermine the quality of maternity services in Australia and threaten the safety of mothers and babies [38]. Discourse generated in this study; 'childbirth is unsafe in the absence of medical expertise', 'a healthy baby is all that matters'. Selected statements illustrating the meanings of these discourses included:

*'Of course, women might feel that sometimes the medical profession interferes too much in what is a natural process, but the reality is that if left to mother nature then the outcomes is not very good often. There needs to be a sensible balance struck between interfering in a natural process but judiciously intervening when things start to go wrong, or preferable before things start to go wrong' (quote article 7)*.

*'The trouble is we take safety for granted now and are arguing about quality issues, like maternal satisfaction, which is important. But I'm sorry not as, as a clinician, survival is the most important thing' (quote article 25)*.

*'If birth is a natural process so is getting cancer and dying' (quote article 12)*.

*'It's a myth to say that the most important thing is a healthy baby. Traumatic birth gets carried with you - you don't leave it at the hospital- and it can have the most profound consequences for both the mother and baby' (quote article 7)*.

On the issue of expanding midwifery -led maternity services as part of the proposed primary maternity care reform agenda some medical and obstetric opinion reported in new articles became divergent as seen in the following statements:

'I fear the introduction of a system where women are pressured into having a natural birth that puts them and their baby at risk' (quote article 18).

'If we can have a patient seeing either the midwife or the GP depending on who is the appropriate person at the time, then that's going to benefit the patients and free the GP's up to be able to see other people. The GP's in rural area's providing birthing services know they can't do it by themselves' (quote article 23.1)

On the issue of planned home birth there is strong resistance reported in news articles from medical experts a position supported in a recently published systematic review of the medical literature on the maternal and newborn safety [39]. Findings are consistent with earlier reports that planned home birth is associated with a tripling of the neonatal mortality rate. The newspaper report failed to clarify whether results included planned home births with experienced midwives in attendance or the controversial practice of free birth in the absence of any professional assistance.

*'Any birth at home under the care of midwives was unsafe and should not be supported by governments (quote article 2)*.

A source of confusion for the general public is evident in reports of obstetric opinion where midwifery services are only recognised as they relate to home birth, failing to acknowledge the work of midwives across the full range of maternity services. Discourse generated in this study; 'midwives act irresponsibly when attending a women birthing at home', 'home birth is a return to times past where mothers and babies died in vast numbers'. Selected statements illustrating the meanings of these discourses included:

*'Midwives do things in a home setting, that no maternity service around the developed world would think a good idea' (quote article 36)*.

*'Home births with or without midwife help, increase the risk of infant death three fold' (quote article 27)*.

### Consumer opinion

The opinions of consumers are varied. Consumers advocating for increasing services supporting natural birth project positivity around women's ability to give birth with minimum intervention in a safe environment. The consumers represented in 'The Age' survey appear fearful, unsure of what is best, placing their trust in health professionals to keep them safe. There is evidence to indicate the presence of the 'what is must be best' belief where the public values the status quo over options for which they have little experience [40,41].

Contrasting discourse were generated in this study; ''women are the experts in giving birth, trust our bodies to keep us safe', 'Australia is one of the safest countries in which to give birth or be born, what is must be best'. Selected statements illustrating the meanings of these discourses included:

'While most mothers were generally satisfied with their experience of the maternity system, hundreds of women felt that both public and private maternity wards are overcrowded, needed more midwives, performed too many unnecessary medical interventions and did not provide enough non-drug options for pain relief' (quote article 18)

*'A lack of obstetricians available after hours means junior registrars are making critical decisions, often bad ones'. I was not supported well enough to have a vaginal birth. I felt like they were more concerned with getting me in and out quickly so they could free up the beds (quote article 19)*.

Consumer opinion reported in newspaper articles on the issue of midwifery -led maternity services including home birth was sought from consumer organisations advocating for non obstetric -led services, supporting women to give birth safely with minimal intervention [25]. Consumer satisfaction with these services was described in glowing terms. Discourses generated in this study include; 'women need to be able to trust their caregivers to act in their best interests, not in the interests of the hospital, doctors or midwives' and 'safe and secure in a relationship built on mutual trust and respect with a known carer', represent the differences in experience between a fragmented maternity service and what can be achieved in a caseload continuity of care or home birth model.

*'Over 400 submissions to the national review from consumers, the majority requesting greater access to home birth cannot be ignored any longer' (quote article 24)*.

*'You seem to have left out the home birth option in your report. Provided the women is healthy, well informed and well supported, there is no reason she cannot give birth at home with the aid of a trusted midwife. My wife did so three times, 25 - 30 years ago (quote article 17)*.

While most of the news articles are concerned with the exciting process of giving birth, consumer opinion raises the issue of private and public hospital care following birth. Traumatic experiences of new mothers left to care for their babies following caesarean surgery are described, as are distressing stories describing lack of support with breast feeding. Discourse generated in this study; 'recovering from the effects of surgery while struggling to mother my baby'. Selected statements illustrating the meanings of these discourses included:

*'Thirty per cent of women in postnatal care wards have had major abdominal surgery and they are expected to try and care for a newborn baby' (quote article 9)*.

*'Nurses are not caring for women after caesareans properly because of overcrowding. Unless you scream the loudest you don't get looked after' (quote article 19)*.

### Midwifery opinion

Expert opinion supplied by the ACM strenuously challenges the obstetric position that only they have the training and expertise to safely care for pregnant women. In each article the midwifery professional body challenges current obstetric practices leading to rising rates of medical intervention and culminating in an alarmingly high rate of caesarean section in healthy young women having their first baby. Discourse generated in this study; 'rates of medical intervention in Australia are too high', 'medical intervention is not without risk it comes with the potential for serious complications'. Selected statements illustrating the meanings of these discourses included:

*'We (midwifery professor) have consistently found that 30% of women report that their birth was traumatic; that they feared for their life or their baby's life. This is a very high figure. We also know that about 6% go on to develop post-traumatic stress disorder' (quote article 7)*.

*'Babies born by caesarean section are more vulnerable to asthma, allergies and infection because they miss out on receiving their mothers' good bacteria during birth. Babies delivered vaginally received protective bacteria as they pass through the birth canal (quote article 8)*.

Reported midwifery opinion reinforced the desirability of midwifery -led maternity services designed to meet the needs of women as mothers. The ability to build a relationship with a known carer based on mutual respect and trust is promoted by advocates of midwives as the panacea for being able to control levels of fear, maintain feelings of being in control throughout the birthing process and being satisfied with the outcome of the experience [42].

## Discussion

The discourses identified and described in this critical analysis of articles published in 'The Age' national newspaper present the general public with a quandary, what to believe? Careful reporting, inclusive of the range of often divergent expert opinion relevant to each news item, leaves the general public and consumers to evaluate the evidence, forming their own conclusions. The right to decide what to think rather than being told what to think reflects the neoliberal values of modern consumerism a situation supported by the Australian government encouraging women to empower themselves through informed decision making regarding maternity care [43]. The rise of consumerism in maternity care has transformed a passive recipient to an active participant in the decision making process, for some, but not all. Consumers advocating for services that promote natural birth with the support of well organised consumer groups have become active participants in decision making regarding future policy direction. The discourse generated in this study; 'women are the experts in giving birth, trust our bodies to keep us safe' reminds us that the majority of pregnant women will give birth safely with minimal need for intervention [44] in a safe environment with the right support. In contrast consumers satisfied with their obstetric care, in the absence of an organised group representing their view, remain passive recipients in the process of reform. In recognition of the absence of a voice in this group "The Age' newspaper attempted to redress the deficit through the conduct of a survey on a popular website for new mothers. The conduct of this survey provided an alternate viewpoint to that represented by organised consumer groups, a viewpoint that has been absent in the processes guiding the direction of maternity care reform, resultant in a concentration of consumer opinion advocating for a move away from obstetric care in healthy pregnancy. Lack of a vocal consumer support organisation advocating for obstetric-led services has diminished the position of medicine and obstetrics in a climate of rising consumer participation.

The general public has been inundated with headline grabbing reports outlining the deficiencies of maternity service. Obstetric practices that are insensitive and a maternity service that is inflexible resulting in women feeling traumatised by the experience of giving birth have been sensationalised. Evidence presented suggests that maternity services are not meeting the expectations of consumers. Consumer discourses identified in this study; 'the interests of women and their babies must be the focus of maternity care, not the system and those who work in it', and 'maternity care options need to be expanded to allow women choice in the care they receive' are reflected in the primary maternity care agenda for change. However expert medical discourses: 'modern medicine knows best and women would be best served to listen to the experts'; 'the demonization of women who choose to give birth naturally', and 'at least you have a healthy baby' convey a very different message positioning women seeking non obstetric -led care options at odds with maternity care providers.

The discourses describing consumer experiences of maternity care in public and private hospitals: 'next please, feeling depersonalised in the queue'; 'feeling vulnerable in the care of a parade of strangers'; 'expected to place blind trust in those who know nothing about me and still feel safe' captures the consumer experience of a fragmented maternity service care and subsequent distress associated with finding themselves in territory they never dreamed possible [45]. Women are reported to be more fearful of the birthing process than ever before a situation accentuated when confronted with a constant stream of unknown maternity care professionals [42]. The ability to build a relationship with a known carer based on mutual respect and trust is reported to control levels of fear and improved levels of satisfaction with the outcome of the experience [42]. It appears from the responses to 'The Age' survey that women are being herded through a system of fear inducing surveillance in the antenatal period without support. Current medical practice that treats every pregnancy with suspicion until proven otherwise perpetuates a disparity between women's perceived risk and their actual risk for complications. This has been described in news articles as antenatal scare.

Despite the poor performance of maternity services on many levels the general public is reassured by Australia's record of safety in childbirth, a success attributed to modern medical advances. A claim contested by the midwifery profession [46] through the midwifery discourses; 'rates of medical intervention in Australia are too high', 'medical intervention is not without risk it comes with the potential for serious complications'. Authoritative medical opinions serve to undermine general public's confidence in the proposed changes to the way maternity care has traditionally been delivered. The fear inducing discourse; 'childbirth is unsafe in the absence of medical expertise' permeates the majority of news reports challenging the premise on which the primary care reforms are based. This discourse infers that midwifery care is not a safe option and in doing so calls into question the reputation of midwives. A further discourse 'midwives act irresponsibly when attending a women birthing at home' serves to discredit the professionalism of the midwives. This is further compounded by the situation where medical or obstetric reference to the practice of midwifery is associated with home birth leaving the impression that midwives only work in this area. The fear inducing discourse 'home birth is a return to times past where mothers and babies died in vast numbers', sends the message to the public that giving birth has been associated with tragic consequences and that the move to hospital birth and medical care is what made the difference.

The discourses of women living in rural and remote communities and strong consumer organisations representing the interests of these women have featured strongly in the proposed reform agenda [31,47-49]. Returning low risk maternity services to local communities is a feature of the change agenda [2]. The discourse 'the lives of mothers and babies in rural communities are placed at risk by widespread closures of maternity services' challenges the government to address a dangerous in-equity in maternity care. The discourse calls into question the widely proclaimed safety of Australian maternity services.

## Conclusion

Findings from this critical discourse analysis revealed that Australian maternity services are being portrayed to the general public as an inflexible outdated service struggling to meet the needs of pregnant women and in desperate need of reform. The style of reporting employed in this newspaper involved presentation to the reader the range of expert opinion relevant to each topic, frequently involving polarised positions of the experts on the issue. As a result the general public are presented with a conflict, caught between the need for the changes that come with a primary maternity model of care and fear that these change will undermine safe standards. The discourse; 'Australia is one of the safest countries in which to give birth or be born, what is must be best', represents the situation where despite major deficiencies in the system the general public may be too fearful of the consequences to consider a move away from reliance on traditional medical -led maternity care.

## Competing interests

The authors declare that they have no competing interests.

## Authors' contributions

MM was responsible for the study conception, design, review and analysis of the literature and drafting of the manuscript. KF and YC made critical revisions to the paper for important intellectual content. All authors have approved the final manuscript.

## Pre-publication history

The pre-publication history for this paper can be accessed here:

http://www.biomedcentral.com/1471-2393/11/47/prepub
